# STAT6 Blockade Abrogates *Aspergillus*-Induced Eosinophilic Chronic Rhinosinusitis and Asthma, A Model of Unified Airway Disease

**DOI:** 10.3389/fimmu.2022.818017

**Published:** 2022-02-23

**Authors:** Hua Sun, Ashish Damania, Megan L. Mair, Eniola Otukoya, Yi-Dong Li, Katherine Polsky, Yuying Zeng, Jeremiah A. Alt, Martin J. Citardi, David B. Corry, Amber U. Luong, John Morgan Knight

**Affiliations:** ^1^Center for Immunology and Autoimmune Diseases, The Brown Foundation Institute of Molecular Medicine for the Prevention of Human Diseases, McGovern Medical School, The University of Texas Health Science Center at Houston, Houston, TX, United States; ^2^Department of Otorhinolaryngology—Head and Neck Surgery, McGovern Medical School, The University of Texas Health Science Center at Houston, Houston, TX, United States; ^3^Department of Pediatrics—Tropical Medicine, Baylor College of Medicine, Houston, TX, United States; ^4^Department of Molecular and Human Genetics, Baylor College of Medicine, Houston, TX, United States; ^5^Department of Medicine, Baylor College of Medicine, Houston, TX, United States; ^6^Division of Pulmonary and Critical Care Medicine, The First Affiliated Hospital of Sun Yat-sen University, Guangzhou, China; ^7^Division of Otolaryngology, Department of Surgery, University of Utah School of Medicine, Salt Lake City, UT, United States; ^8^Department of Pathology and Immunology, Baylor College of Medicine, Houston, TX, United States; ^9^Michael E. Debakey VA Center for Translational Research in Inflammatory Diseases, Houston, TX, United States

**Keywords:** STAT-6, allergic, mycosis, asthma, chronic rhinosinusitis, mouse model, unified airway, eosinophil

## Abstract

Unified airway disease, including concurrent asthma and chronic rhinosinusitis (CRS), is a common, but poorly understood disorder with no curative treatment options. To establish a murine model of chronic unified eosinophilic airway inflammation, mice were challenged with *Aspergillus niger*, and sinonasal mucosa and lung tissue were evaluated by immunohistochemistry, flow cytometry, and gene expression. Inhalation of *A niger* conidia resulted in a Th2-biased lung and sinus inflammation that typifies allergic asthma and CRS. Gene network and pathway analysis correlated with human disease with upregulation of not only the JAK-STAT and helper T-cell pathways, but also less expected pathways governing the spliceosome, osteoclast differentiation, and coagulation pathways. Utilizing a specific inhibitor and gene-deficient mice, we demonstrate that STAT6 is required for mycosis-induced sinus inflammation. These findings confirm the relevance of this new model and portend future studies that further extend our understanding of the immunopathologic basis of airway mycosis and unified airway disease.

## Introduction

Broadly, chronic rhinosinusitis (CRS) describes a chronic upper airway inflammation condition. The inflammatory response can be characterized by an influx of immune cells into the sinonasal tissue, goblet cell hyperplasia, and subepithelial thickening resulting in symptoms of facial pain and pressure, nasal congestion, and persistent drainage that affects approximately 3%–6% of the US population (1). Sharing similar immune and histologic changes to CRS associated with elevated type 2 immune profile, asthma is a comorbid disease in 50%–70% of CRS patients (2). In addition, both are linked to a dysregulated interaction between respiratory epithelial cells and the immune system in response to exposures to environmental triggers including fungi.

Severe asthma and CRS with nasal polyps (CRSwNP) show strong correlations with T cell and antibody-specific sensitization to fungi such as *Alternaria alternata* and *Aspergillus niger* (3, 4). Mice exposed to *A. niger* develop airway hyperresponsiveness, the physiologic *sine qua non* of asthma, increased production of the type 2 cytokines IL-4, IL-5, and IL-13 in the lungs, along with an influx of eosinophils and elevated serum IgE levels (5). Several mouse models for CRS have been proposed (**Supplementary Table 1**), but lack widespread acceptance given the inclusion of non-physiologic exposures and shortcomings in sinonasal analysis that provide insufficient validation (6, 7). Thus, despite a pressing need for a mouse model of CRS for pathophysiology, etiologic, and preclinical studies, no model has been widely adopted.

As of 2019, a monoclonal antibody targeting IL-4 and IL-13, dupilumab, which has indications for moderate-to-severe eosinophilic asthma, was FDA approved for CRSwNP. Although efficacious for both asthma and CRS, dupilumab and other biologics are expensive (ranging from $10,000 to $40,000 per year) and noncurative. The efficacy of dupilumab in the treatment of severe allergic asthma and CRS and studies in STAT6-deficient mice highlight the central role of the IL4/IL13-IL4Rα-STAT6 signaling pathway in developing eosinophilic asthma (8–10). Additionally, our group has pioneered the emerging paradigm that fungi are fundamental causes of both asthma and CRS (4, 5, 11) through the elaboration of key virulence factors such as proteinases (12), cytokines [cleaved fibrinogen (13, 14)], and candidalysin (15). Using an *A. niger* challenge-based mouse model of allergic asthma, STAT6-deficient mice failed to develop airway hyperresponsiveness and pulmonary eosinophilia (16). Moreover, mimetic peptides targeting the SH2 domain of STAT6 have been developed that block the function of both STAT5 and STAT6 (17). These aerosolized small-molecule inhibitors, which minimize systemic adverse effects, not only inhibited the development, but also reversed airway mycosis-dependent allergic lung disease in preclinical allergic airway disease (10). Given the lack of a widely accepted mouse model for CRS, the utility of these promising small peptide inhibitors and other potential therapeutics has not been adequately evaluated for chronic eosinophilic sinus inflammation. Here, we describe a new mouse model of eosinophilic upper and lower airway inflammation that is based on the induction of airway mycosis. Herein, we extensively validate this fungus-driven model with respect to established gene induction patterns in human CRS and asthma and further evaluate the central role of STAT6.

## Methods

### Mice

C57BL/6 and Stat6^-/-^ mice (4 weeks old females) were purchased from The Jackson Laboratory (Bar Harbor, ME).

### Stat6 Inhibitor

The STAT6 inhibitor is formulated in liposomes with 1,2-dilauroyl-sn-glycero-3-phosphocholine (DLPC; 850335, Avanti Polar Lipids) as previously described (10). Prior to formulating daily treatments, vehicle and drug suspensions were briefly sonicated for 30 s and vortexed.

### Eosinophilic Fungal Murine Model

Mouse intranasal challenge was performed as previously described (5). Briefly, 4 × 10^5^ A*. niger* conidia were suspended in sterile phosphate-buffered saline (PBS) in a total volume of 50 μl (8 × 10^6^/ml). The fungal suspension was intranasally (i.n.) instilled into each mouse every other day for 2 weeks or 3 months. Control mice were instilled with 50 μl of PBS. For Stat6 inhibitor experiments, mice received daily i.n. treatment with vehicle (DLPC) or 10 ng of Stat6 inhibitor suspended in 50 µl of PBS, or fungal suspensions every other day.

### Airway Physiology

Respiratory system resistance (R_RS_) in response to increasing doses of acetylcholine was measured by whole-body, semi-invasive plethysmography, as previously described (5).

### Histological Analysis

The mice for histological analysis were decapitated and the skulls were fixed in 10% neutral-buffered formalin. The lungs were perfused and fixed in 10% neutral-buffered formalin as well. Tissue samples were submitted to the Pathology Core, at Baylor College of Medicine, for processing (decalcification and imbedding) and serial 5-μm-thick coronal sections on skull samples and 5-μm-thick sections on lung tissues were prepared. Both tissues were subjected to hematoxylin and eosin (H&E) and Periodic acid–Schiff (PAS) stain. The slides were analyzed by two examiners blinded to the group assignments.

### Mouse Nasal Lavage Fluid Analysis

The mice were decapitated and a 24-gauge catheter tube was inserted into the tracheal orifice and lavage two times of 150 μl of sterile PBS. The two times fluid from one mouse was combined, resuspended into 150 μl of PBS, and cytospun prior to May-Giemsa staining using Diff-Quik stain set (Dade Behring, DE). Cells were counted and differentiated by two examiners.

### Mouse Sinonasal Epithelial Cells Isolation

The mice subjected to lavage were used for sinonasal epithelial cells isolation. The sinonasal cavity was opened by removing the nasal and frontal plates, septum was disassociated, and tissue was scraped out with forceps into a 1.5-ml microcentrifuge tube and digested by 400 μl of dissociation buffer (1× PBS, 5% BSA, 10 mM HEPES, 5 mM KCl, 1 mM MgCl_2_, 1.8 mM CaCl_2_, 50 U DNase I, and 2 mg/ml Collagenase D) for 30 min. Single-cell suspensions were flushed through the 70-μm cell strainer and washed with 10 ml of DMEM medium with 10% FBS, 100 U/ml penicillin, and 50 μg/ml streptomycin. The cells were resuspended in PBS for flow staining or 200 μl of PBS containing 1% FBS for sorting. The epithelial cells were sorted by FITC-labeled anti-EpCAM (G8.8, BioLegend) and cultured in complete media.

### Flow Cytometry Sorting and Analysis

Single-cell suspension from sinonasal tissues and lungs were Live/Dead stained (Zombie NIR; BioLegend), blocked with anti-CD16/CD32 (clone 93, BioLegend), stained for inflammatory cells with CD45-PE (103106, BioLegend), Siglec-F-BV510 (740158, BD), CD125-BV650 (740623; BD), CD62L-APC (553152, BD), CD64-BV605 (139323, BioLegend), Ly-6G-PE-CF594 (562700, BC), TCRβ-PE-Cy7 (109222, BioLegend), and CD4-BUV737 (749058, BD) in FACS buffer (PBS with 0.05% BSA), and resuspended in FACS buffer for sorting or FACS Lysing buffer (349202, BD) for analysis. For intracellular cytokine staining, single-cell suspensions were incubated with 50 ng/ml phorbol 12-myristate 13-acetate (PMA, Biogems), 500 ng/ml ionomycin (Biogems), and Brefeldin A solution (BioLegend) for 4 h at 37°C with 5% CO_2_. The cells were Live/Dead stained, Fc receptor was blocked, and surface markers were stained [CD3-BV750 (100232, BioLegend) and CD4-FITC (100406, BioLegend)] and permeabilized with Cytofix/Cytoperm Plus Kit (BD Biosciences) for intracellular staining of IFN-γ-PE-Cy7 (505826, BioLegend), IL-5-APC (504306, BioLegend), IL-13-eF450 (48-7133-82, ThermoFisher), and IL-17A-BV650 (506930, BioLegend). To quantify cells, CountBright beads (C36950, ThermoFisher) were added to samples prior to analysis on an LSRII cytometer (BD) or sorting/analysis on an Aria-II (BD) and FlowJo 10.4 software (Tree Star, Inc., Ashland, OR).

### RNA Extraction and Sequencing

Total mRNA from scraped non-lavaged sinus tissue or sorted mouse sinonasal epithelial cells were isolated with RNeasy Mini Kit (Qiagen, Venlo, Netherlands). Tissue RNA was submitted to the Genomic & RNA Profiling Core at Baylor College of Medicine for quality control and library preparation. Qualified samples were sequenced on an Illumina NovaSeq Platform.

### Processing of RNA-Seq Data

Paired-end fastq reads were filtered with fastp (v0.21.0) set at a minimum length of 50 bp and a minimum Phred score of 15. Filtered fastq reads were mapped to mouse genome (Gencode release M25) with STAR aligner (v2.7.7a) with RSEM (v1.3.3) and differentially expressed gene (DEG) analysis with DESeq2 (v1.30.1). Differential isoform usage was determined with Dexseq (v1.36.0) using IsoformSwitchAnalyzeR (v1.12.0). Figures and analysis were generated using R (R Core Team, 2020) with ggplot2 (v3.3.3) and ggsashimi (v0.6.0).

### Gene Signature Analysis

Human asthma epithelial gene signature was derived from the Gene Expression Omnibus (GEO) microarray datasets (GSE18965, GSE43696, and GSE9150), aggregated for significantly (*p*_adj_ < 0.05) upregulated genes in unstimulated asthma vs. healthy control samples. Similarly, human CRS gene signature was aggregated from published datasets by Alt et al. (submitted) and Luong et al. (18). Overlap between asthmatic human airway epithelial cells (hAEC), murine asthma (19) and murine CRS, and human CRS and murine CRS gene signatures was performed in R. KEGG pathway enrichment analysis on DEGs, gene signatures, and overlapping gene sets was performed with pathfindR (v1.6.2), filtered for significant enrichments (*p*_adj_ < 0.01), annotated for KEGG pathway map category (Metabolism, Signal Transduction, etc.), and visualized using ggplot2 and Cytoscape (v3.7.1).

### Targeted Gene Expression

Total sinus tissue and sorted epithelial cell cDNAs were synthesized with iScript cDNA synthesis kit (Bio-Rad, CA). Taqman gene expression master mix and Taqman probes (Applied Biosystems) for each gene were used and normalized to 18S rRNA. Gene expression was calculated using the ΔΔCt method relative to naïve samples.

### Statistical Analysis

Data were analyzed with GraphPad Prism software (version 6). Comparisons of two groups were calculated with two-tailed unpaired *t* test, unless specified as one-tailed. Comparison of more than three groups was calculated with one-way or two-way analysis of variance (ANOVA) with Tukey’s test. Differences with *p*-values below 0.05 were considered significant. Isoform usage was analyzed with IsoformSwitchAnalyzeR with default settings, and differences with *q*-values below 0.05 were considered significant. Hypergeometric analysis was performed with a genomic population size *N* = 55,401 for total number of genes potentially mapped in Gencode release M25.

### Study Approval

All experimental procedures and handling of the mice were reviewed and approved by the Institutional Animal Care and Use Committee of Baylor College of Medicine and followed all federal guidelines.

## Results

### Intranasal *A. niger* Exposure Results in Eosinophilic Sinus and Pulmonary Airway Inflammation and Upregulation of Th2 and Th1 Inflammatory Signaling Pathways

**Figure 1:**
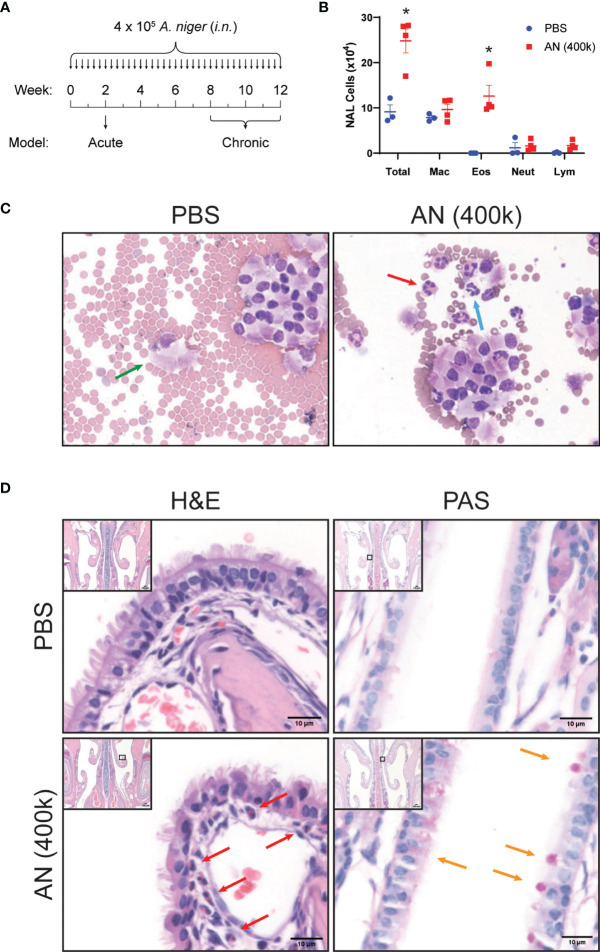
Intranasal fungal challenge promotes eosinophilic sinus inflammation. **(A)** Experimental outline of acute and chronic models. Mice were i.n. challenged with 4 × 10^5^ *A. niger* (AN) or PBS every other day for 2 weeks (acute) or 2–3 months (chronic) and assessed for inflammation. **(B)** Nasal airway lavage (NAL) inflammatory cells were quantified **(C)** from H&E-stained slides. **(D)** Representative H&E (left)- and PAS (right)-stained sinus sections from PBS- and AN-treated mice. Arrows indicate epithelial cells (green), eosinophils (red), neutrophils (blue), and mucus-stained goblet cells (orange). Results represent mean ± s.e.m. from two independent experiments; *p < 0.001, *n* ≥ 3 by two-way repeated measures ANOVA.

We challenged mice with *A. niger* (AN) conidia intranasally for 2 weeks and evaluated nasal lavage fluid and sinonasal mucosa for inflammatory changes (**Figure 1A**). As assessed by nasal lavage, chronic fungal exposure led to a pronounced influx of eosinophils, but not neutrophils or lymphocytes, as compared to vehicle challenged animals (**Figure 1B**). Histopathologic analysis of the sinonasal mucosa of these mice confirmed the presence of mucosal interstitial eosinophils and airway epithelial goblet cells in fungus, but not vehicle, challenged animals (**Figures 1C, D**). These findings are consistent with the allergic inflammation that is typically seen in human fungus-associated CRS (20).

In addition, we confirmed that after 2 weeks of intranasal fungal airway exposure, mice develop airway hyperresponsiveness together with prominent interstitial eosinophilia and goblet cell metaplasia (**Supplementary Figure 1**). We have previously characterized lung inflammation in mice following 1–2 weeks challenge with fungal proteinase, candidalysin, cleaved fibrinogen, and live fungi (5, 12, 14). All of these challenges induce a stereotypical pattern of lung physiologic and inflammatory changes that include the induction of exaggerated contractile responses of the airway with acetylcholine challenge (airway hyperresponsiveness), enhanced lung and airway eosinophilia, and goblet cell metaplasia of the lower airways. Thus, mice challenged intranasally with the conidia of *A. niger* simultaneously develop allergic inflammation of the upper and lower airways.

**Figure 2:**
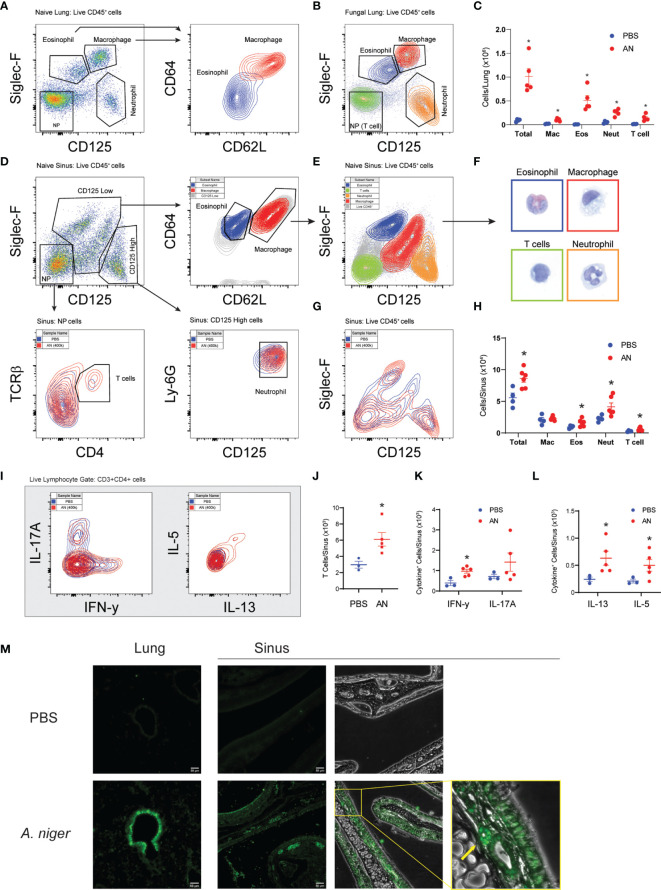
Airway mycosis induces eosinophilic, TH2-high inflammation in the upper and lower airways. Mice were challenged with 4 × 10^5^ *A. niger* (AN) or PBS daily for 2 months. Representative flow plots of lung single cell suspensions from **(A)** PBS or **(B)** fungal challenged (AN) wild-type C57BL/6 mice, with gates for eosinophils (Eos), macrophages (Mac), neutrophils (Neut), and TCRβ+CD4+ T cells in the negative population (NP), and sub-gates for Eos/Mac. **(C)** Differential cell counts from lungs. **(D)** Representative flow plots of naïve sinus, showing gating scheme for differential cell analysis and **(E)** sorting of cells for direct **(F)** imaging of H&E-stained cells (border colors match sorted population). **(G)** Overlay of fungal and PBS-treated mice and **(H)** differential cell counts of sinus inflammatory cells. **(I)** Representative flow plots of *ex vivo* stimulated TCRβ+CD4+ T cell cytokine production from sinuses was analyzed, with **(J)** total T cells, **(K)** IFN-γ+ and IL-17+, and **(L)** IL-13+ and IL-5+ cells quantified. **(M)** Fluorescent immunohistochemical staining of mouse sinus after PBS and *A. niger* challenge showing distribution of major basic protein, yellow box and arrow highlighting an eosinophil. Results presented as mean ± s.e.m. from at least two independent experiments with *n* = 3-5. *p < 0.05, *n* ≥ 3; by two-way ANOVA for differential counts and one-tailed *t*-test for cytokine production.

Next, we conducted flow cytometry-based analyses of airway cells to characterize in detail the inflammation induced by chronic (12 week) intranasal exposure to *A. niger* conidia (**Figures 2A**–**L**). We developed a protocol that allowed us to identify and quantify macrophages, eosinophils, neutrophils, and T cells from CD45+ cells obtained from both lung (**Figures 2A**–**C**) and sinus (**Figures 2D**–**H** and **Supplementary Figure 2**) tissues of the same mice (21). These studies demonstrated that, similar to mice challenged intranasally with *A. niger* over 2 weeks (5), chronic airway fungal exposure results in elevated total lung inflammatory cells composed of significantly increased macrophages, eosinophils, neutrophils, and T cells as compared to vehicle challenged animals (**Figure 2C**). Moreover, we found that the increased sinus mucosal total cells and eosinophils seen after 2 weeks of fungal exposure (**Figure 1**) persisted out to 12 weeks (**Figure 2H**). Additionally, we found that chronic fungal exposure resulted in elevated neutrophils and T cells in sinonasal mucosa (**Figure 2H**). Neutrophils were the largest contributor to this increase, presumably enhanced by inclusion of interstitial cells as compared to sinus lavage alone (**Figure 1B**). Thus, unlike the allergen ovalbumin that provokes acute eosinophilic airway inflammation that gradually wanes with chronic exposure (22), chronic exposure to *A. niger* conidia results in sustained and even progressive eosinophilic sinus inflammation over chronic exposure periods.

We next determined the immune phenotype of CD4+ T cells infiltrating the sinonasal mucosa of mice chronically challenged with *A. niger* using intracytoplasmic cytokine staining (**Figures 2I**–**L**). These findings confirmed the enhanced recruitment of T cells to sinus tissue after fungal challenge (**Figure 2J**) and demonstrated the significant recruitment of both (IFN-γ-expressing) Th1 cells (**Figure 2K**) and (IL-5 and IL-13-expressing) Th2 cells (**Figure 2L**), a T-cell phenotype that parallels what has been previously described for lung T cells from *A. niger* challenged mice (5). However, whereas (IL-17A-expressing) Th17 cells are prominent in acute *A. niger* challenged mouse lung (23), we did not find a statistically significant increase in sinus Th17 cells after chronic *A. niger* exposure, although some mice did exhibit enhanced sinonasal Th17 cells (**Figure 2K**). To confirm the presence of eosinophils in mouse sinus tissue, we performed immunofluorescence microscopy for the major basic protein (MBP), a major secreted protein of eosinophils, after *A. niger* challenge (**Figure 2M**). As compared to PBS challenged mice, *A. niger* challenged mice demonstrated robust, diffuse staining of MBP along the airway epithelium and surface mucus layer and within luminal cells within both the sinuses and lungs. Together, these findings demonstrate that chronic intranasal *A. niger* challenge results in (1) persistent eosinophilic inflammation marked by the robust recruitment of both Th1 and Th2 cells to sinonasal mucosa and (2) MBP is secreted diffusely by eosinophils within the mouse sinonasal mucosa after *A. niger* challenge.

### *A. niger* Challenge Activates Overlapping Immune and Signaling Pathways as Seen in Human CRS and Allergic Asthma

**Figure 3:**
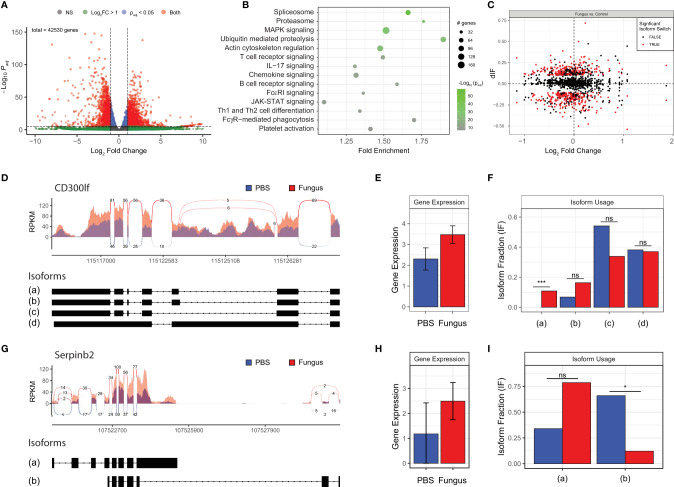
Fungal-mediated enrichment of inflammatory pathways of differentially expressed genes (DEGs) reveals variable gene isoform usage. Mice were i.n. challenged with *A. niger* (AN) or PBS as in **Figure 1A**, total RNA was isolated from sinus, and RNA-seq analysis was performed. **(A)** Volcano plot of DEGs in fungal challenged mouse sinus tissue vs. vehicle in which gene expression is depicted as fold change versus adjusted *p*-value (log10). **(B)** KEGG enrichment of significant pathways, with color indicating statistical significance and circle size indicating the number of genes. **(C)** Isoform switch analysis of fold change in sequenced gene isoforms for fungal challenged mice vs. naïve controls (dIF), with significantly switched isoforms shown in red. CD300lf **(D–F)** and Serpinb2 **(G–I)** sashimi plots of read coverage with isoform exon maps **(D, G)**, total gene expression **(E, H)**, and isoform usage **(F, I)**. *p*_adj_ < 0.05 and *q* < 0.05; *n* = 3 by Student’s *t*-test. *p < 0.05; ***p < 0.001; ns, not significant.

We conducted extensive additional studies to characterize how gene expression evolves in response to airway mycosis of the mouse sinuses. RNA sequencing analysis (RNA-seq) of total RNA extracted from mouse sinuses after 12 weeks of fungal challenge revealed numerous highly significant differences in DEGs, including DEGs that were both higher and lower in expression in mice with airway mycosis as compared to vehicle challenged (**Figure 3A** and **Supplementary Table 2**). KEGG enrichment analysis of DEGs revealed that many canonical inflammatory and related pathways were activated in mouse sinus tissue in response to chronic fungal exposure (**Figure 3B**). Consistent with the predominant Th1 and Th2 cytokine pattern observed from the same tissues (**Figure 2**), we found prominent upregulation of signaling pathways controlling T-cell receptor signaling, Th1 and Th2 differentiation, JAK-STAT signaling, and many others. Intriguingly, although IL-17 was not significantly induced in sinus T cells (**Figure 2K**), genes controlling IL-17 signaling were significantly induced.

Our DEG analysis further revealed major changes in expression of genes associated with ubiquitin-mediated proteolysis and the proteasome, highlighting significant cellular activity and protein production and turnover (**Figure 3B**). In addition, spliceosome components responsible for mRNA precursor processing and generation of isoforms were induced within the sinonasal mucosa of our mouse model of eosinophilic CRS (**Figures 3C**). Detailed analysis of selected spliced genes revealed that even where overall gene expression does not differ, fungal challenge results in differential expression of specific isoforms [e.g., isoform (a) of CD300lf; **Figures 3D**–**F**] and marked downregulation of major isoforms during airway mycosis [e.g., isoform (b) of Serpinb2; **Figures 3G**–**I**].

**Figure 4:**
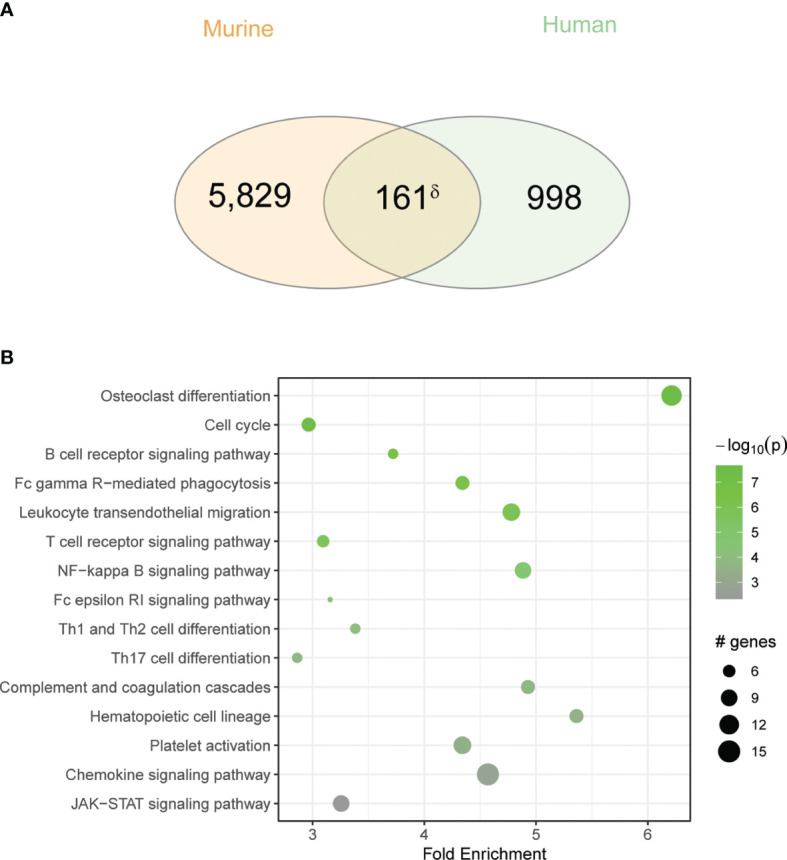
Overlap between human CRS and chronic fungal challenged mouse sinus gene expression is enriched for inflammatory pathways. **(A)** Venn diagram showing the overlap between DEGs in the murine model to the CRS gene signature. **(B)** KEGG pathway enrichment analysis of overlapping genes. δp < 5e-10 by hypergeometric analysis.

To extend these findings to a more clinically relevant context, we next compared gene expression in inflamed sinus mucosa between our fungal-induced murine model of eosinophilic sinusitis and human CRS (**Figure 4**). We identified 161 overlapping genes that are shared between mouse and human tissues (**Figure 4A**). KEGG pathway analysis of these shared genes revealed consistent enhancement of pathways involved in T-cell differentiation and inflammatory signaling including the JAK-STAT pathway (**Figure 4B**). Additionally, this analysis revealed the enhanced expression of less explored pathways including osteoclast differentiation, related genes of which constituted the largest number of genes upregulated with the greatest fold enrichment relative to other pathways (**Figure 4B**). Of note, osteitis and osteoneogenesis are frequently seen in CRSwNP and are associated with disease severity and recurrence (24). Thus, key inflammatory and related genes are upregulated and signaling pathways are operative to a similar degree in mice with airway mycosis-dependent eosinophilic CRS and human CRS.

**Figure 5:**
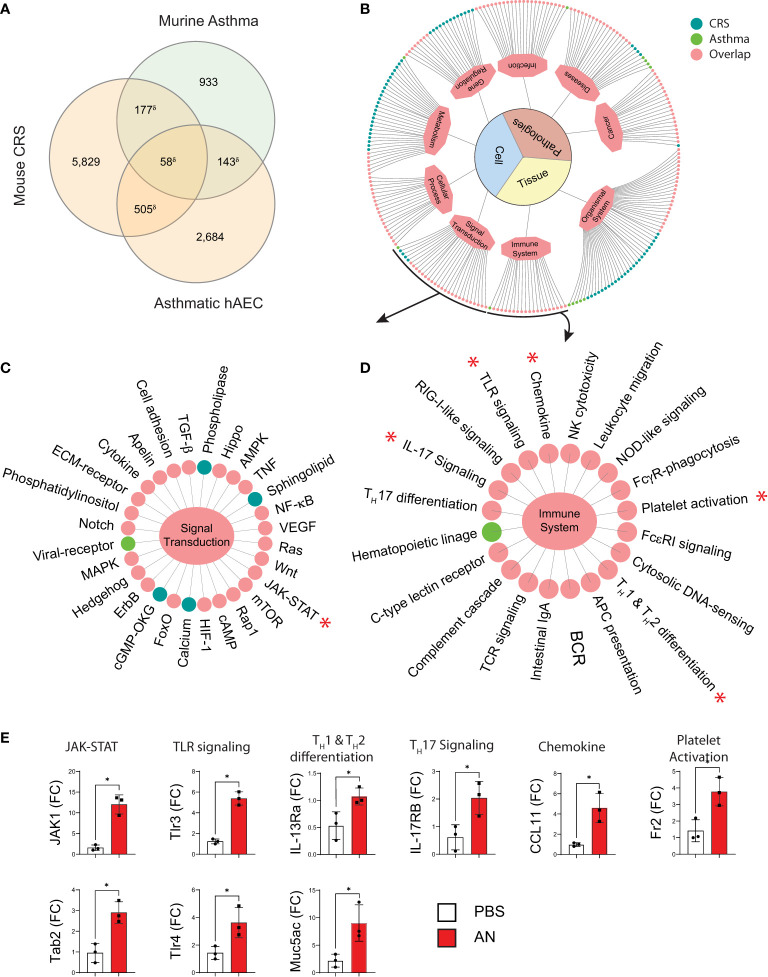
Murine fungal rhinosinusitis has an asthmatic inflammatory gene signature. **(A)** Venn diagram showing overlap between DEGs and the asthma signature gene set for murine asthma21 and asthmatic human airway epithelial cells (hAEC). **(B)** KEGG pathway enrichment network and sub-networks including **(C)** Signal Transduction and **(D)** Immune System, for sinus DEGs (CRS; green), murine asthma (Asthma; blue), and co-enriched (Overlap; pink) pathways. **(E)** qPCR validation of select genes in co-enriched pathways (*) from sinus total RNA. δp < 5e-15 by hypergeometric analysis; *p*_adj_ < 0.05 for KEGG pathway enrichment; *p < 0.05, *n* = 3 by Student’s *t*-test.

We discovered significant homology with respect to gene expression when we compared transcriptomic profiles of our mouse CRS model, pulmonary tissue from a murine allergic airway disease model, and human airway epithelial cells from asthmatic patients (**Figure 5A**). KEGG analysis revealed numerous biologically relevant pathways from murine airway transcriptomic samples that included immune, cellular, infection, and cancer pathways (**Figure 5B**). Focusing specifically on pathways associated with signal transduction and immune pathways, this analysis again revealed homologous activation of JAK-STAT, TLR signaling, chemokine signaling, and Th1 and Th2 differentiation pathways (**Figures 5C, D**). Although these findings are limited by the non-specificity of the pathways, we confirmed upregulation of select genes from representative pathways by RT qPCR (marked by asterisks; **Figure 5E**). Thus, similar immune signaling pathways are operative in diverse eosinophilic inflammatory contexts that include mouse lungs, human airway epithelium, and mouse sinus.

### Upregulated Expression of Alarmins, Antimicrobial Peptides, and Asthma-Linked Genes in Sinonasal Epithelial Cells During Murine Airway Mycosis

**Figure 6:**
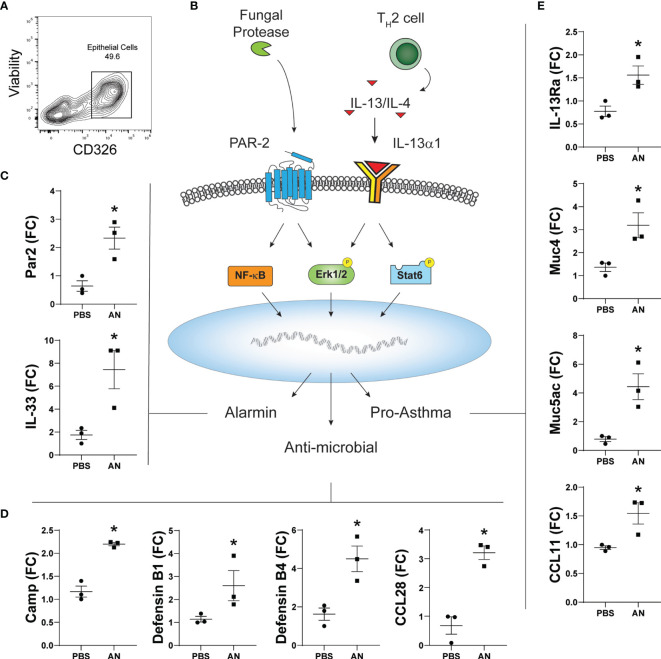
Chronic airway mycosis promotes sinus epithelial cell alarmin, anti-microbial, and pro-asthma gene expression. **(A)** EpCam-positive (CD326+) epithelial cells were sorted from mice chronically challenged with 4 × 10^5^ *A. niger* (AN) or PBS every other day for 2 months for gene expression. **(B)** Diagram of relevant epithelial responses to fungal protease and Th2-driven inflammation. Protease activates protease activated receptor 2 (PAR-2) signaling to NF-κB and Erk1/2 to induce **(C)** alarmin and **(D)** antimicrobial gene expression. TH2 cytokines IL-13 and IL-4 signaling through IL-13Rα1 activate Erk1/2 and Stat6 to promote **(E)** pro-asthma gene expression. *p < 0.05, *n* = 3 by Student’s *t*-test.

Respiratory epithelial cells play central roles in innate immunity as the initial responders against exposure to environmental pollutants and microbes and in orchestrating immune cells to prevent pathogen invasion (25). Although primarily consisting of ciliated respiratory epithelial cells, chronic environmental exposures associated with asthma and CRSwNP lead to remodeling of the airway epithelium and a disequilibrium in epithelial cell subtypes. Airway basal cells, which are typically quiescent, become activated and increase in numbers upon environmental injury. In asthma and CRS, basal epithelial cell subsets contribute to the innate immune response through the induction of numerous gene classes (26). We therefore compared gene expression from FACS-sorted EpCAM+ epithelial cells from the sinonasal mucosa of PBS and *A. niger* challenged mice (**Figure 6A**). Using RT-qPCR, we determined the relative expression of genes previously linked to fungal proteinase-dependent activation of proteinase activated receptor 2 (PAR-2) and the IL-13 receptor that include alarmins, antimicrobial peptides, and asthma-related genes that are expressed under the control of multiple transcription factors linked to these immune receptors (**Figure 6B**). We found the expected increased expression of not only IL-33 but also PAR2, the expression of which was previously shown for sinonasal epithelial cells from CRS patients (27), suggesting an auto-feedback loop regulating expression of this receptor (**Figure 6C**). Multiple antimicrobial peptides (AMPs) including defensins, cationic antimicrobial peptide (CAMP), and the chemokine CCL28 were also strongly induced (**Figure 6D**). Finally, fungal challenge resulted in the significant upregulation of type 2 associated inflammatory genes including CCL11, IL-13Rα, and the major airway mucin genes Muc5AC and Muc4 (**Figure 6E**). Thus, in addition to AMPs, fungal challenge results in the induction of numerous genes previously linked to asthma and CRS in airway epithelial cells, supporting the critical role these cells play in orchestrating the overall immune response to airway mycosis.

### STAT6 Is Essential for Eosinophilic Upper and Lower Airway Inflammation

**Figure 7:**
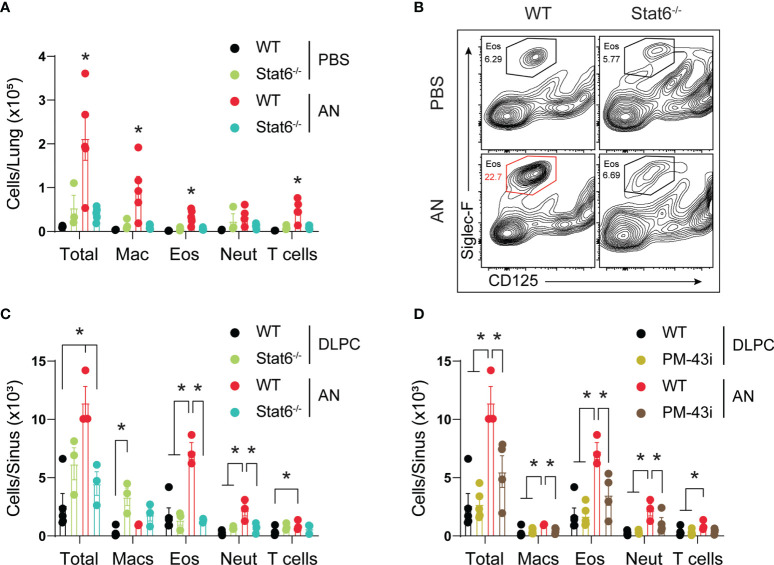
STAT6 is essential for eosinophilic murine sinusitis. **(A)** Stat6-/- or C57BL/6 mice were challenged (i.n.) with 4 × 10^5^ *A. niger* (AN) or PBS for 3 months and lungs were assessed for inflammatory cell recruitment. **(B)** Representative flow analysis of Siglec-F and CD125 expression in wildtype (Wt) and Stat6-/- mice challenged with fungus (AN) or vehicle (PBS/DLPC) and **(C)** quantified. **(D)** Sinus inflammatory cell analysis from fungal challenged mice treated with 10 ng of PM-43i or vehicle (PBS/DLPC). *p < 0.05, *n* ≥ 3 by ANOVA. Data are from one of three representative experiments.

A critical task suggested by our analyses is determining the essential inflammatory pathways that drive eosinophilic inflammation of both the upper and lower airways during airway mycosis. STAT6 has previously been shown to drive eosinophilic lower airway inflammation under a variety of short-term inflammatory challenge conditions, including fungal (10). Utilizing STAT6-deficient mice, we found that challenge of mice with *A. niger* over 12 weeks resulted in a similarly marked reduction in lung eosinophils as assessed by flow cytometry, but we also found near-complete suppression of all other inflammatory cells including macrophages, neutrophils, and T cells (**Figure 7A**). Despite the apparent lack of immune response against a potentially deadly pathogen, STAT6-deficient mice did not lose weight or appear ill in comparison to any other treated group (data not shown).

We further determined the evolution of sinonasal inflammation by flow cytometry during chronic fungal challenge (**Figure 7B**). Comparing STAT6-deficient mice (**Figure 7C**) and wild-type mice treated with a STAT5/6 small peptide inhibitor (PM-43I; **Figure 7D**) shown to reverse established allergic asthma after 4 weeks (10), we found that lack of STAT6 in both contexts suppressed or abrogated sinonasal inflammation, including the reduction of all major inflammatory cells, elicited by *A. niger* challenge. Although STAT6 deficiency was largely protective, topical inhibition of STAT6 significantly attenuated inflammation. These findings confirm the importance of STAT6 to the induction of acute eosinophilic lower airway inflammation, but extend this observation by demonstrating that STAT6 is also generally essential for chronic, persistent inflammation of both the upper and lower airways in the context of airway mycosis.

## Discussion

The unified airway disease concept posits that rhinitis/sinusitis and asthma frequently occur together and, by extension, that upper and lower airway eosinophilic diseases are pathophysiologically related and amenable to similar therapies (28). Consistent with these concepts, we and others have previously linked CRS and asthma to airway mycosis and multiple studies now indicate that aggressive management of CRS is essential to gaining effective control of asthma (4, 11, 29–34). However, an experimental system that rigorously models unified airway disease has heretofore been elusive. By administering conidia of *A. niger* intranasally to wild-type mice over 12 weeks, we have developed a model of eosinophilic CRS with concurrent eosinophilic lower airway disease. Although potentially degraded by the rigors of processing (e.g., decalcification), histologic analysis of sinus tissue showed increased eosinophilia and goblet cell metaplasia. Additionally, flow cytometry and cell sorting—though sorted cells suffer from significant degradation due to the rigors of isolation and processing—analyses of the murine sinonasal mucosa were consistent with inflammatory changes previously noted in human eosinophilic CRS, demonstrating a disorganized respiratory epithelial cell layer, increased goblet cell hyperplasia, and the influx of eosinophils, neutrophils, and T-cell subtypes (35–37). Gene network and pathway analysis along with comparisons to human microarray data and asthma gene datasets not only identified the upregulation of key inflammatory pathways including those linked to JAK-STAT signaling and Th1 and Th2 cell differentiation, but also highlighted less expected pathways governing the spliceosome, osteoclast differentiation, coagulation, and platelet activation. Utilizing this airway mycosis-dependent model of chronic eosinophilic sinusitis and allergic asthma, we previously demonstrated that STAT6-deficient mice and an intranasally applied small peptide inhibitor of STAT6 not only ameliorate pulmonary inflammation and airway hyperreactivity, but also now show that it essentially abrogated sinus inflammation and the influx of inflammatory effector cells to the sinonasal mucosa (10). These findings confirm the relevance of this new model and portend future studies that further extend our understanding of the pathophysiology of airway mycosis and unified airway disease.

Respiratory epithelial cells play an active role in initiating and orchestrating the innate and adaptive type 2 inflammatory response. We have developed techniques to isolate mouse sinonasal mucosa that are suitable for RNA sequencing, flow cytometry with cell sorting, and tissue culture studies. Isolating EpCAM-positive sinonasal epithelial cells, we found that *A. niger* induced expression of the alarmin IL-33, several antimicrobial peptides, and numerous downstream genes of type 2 inflammation such as Muc5AC and chemokine. The inflammatory response between the sinus and lungs was strikingly consistent, hold for the increased abundance of neutrophils in the fungal challenged sinus potentially due to inherent structural differences between the tissues. Sinus epithelial or stromal cells—present in the increased interstitial space of the sinuses—may support the recruitment of neutrophils on top of the classically recognized eosinophilia associated with allergic airway disease. Importantly, loss of Stat6 or partial inhibition of epithelial Stat6—by topical treatment with a Stat6 inhibitor—abrogated eosinophil and neutrophil recruitment in the airway. Given the urgent, largely unmet need to simultaneously treat CRS and asthma with topical therapeutics, understanding the role of respiratory epithelial cells in the initiation and propagation of underlying mucosal inflammation is critical and now becomes feasible utilizing this model.

Although other mouse models of eosinophilic CRS have been proposed, there are some key advantages to our model. First, the lungs of *A. niger* challenged mice are characterized by eosinophilic allergic airway inflammation that strongly resembles findings in allergic asthma. In CRSwNP, the prevalence of asthma is reported to be 48%–73% (2, 38). The prevalence by criteria of aspirin exacerbated respiratory disease, a clinical CRSwNP subtype, and asthma is 100%. However, for allergic fungal rhinosinusitis, another clinical CRSwNP subtype, the prevalence of asthma is notably less, approximately 20%–25% (38), slightly above the prevalence in the general population, but significantly lower than that reported in other CRSwNP subsets. Our mouse model will allow future studies to dissect pathways that may be important in the unified and divergent development of type 2 inflammation between the sinus cavities and lungs.

Secondly, intranasal challenge with fungal conidia over 3 months represents a physiologically relevant exposure for the development of chronic respiratory inflammation. Other mouse models for CRS have been proposed, but fail to recapitulate the environmental exposures experienced by typical patients, e.g., by utilizing non-physiologic means to boost an immune response including intraperitoneal injections of fungi with or without adjuvant (6), use of purified proteases not typically inhaled from the environment (39), or short exposure times of less than 2 weeks (40). In contrast, our model of unified airway disease employs physiological exposure to inhaled conidia over an extended time period to more closely align with the development of chronic inflammation that is typical of CRS.

Dysregulation of the coagulation cascade has been linked to many diseases, including asthma and sinusitis (41–43). Fibrin, the major end product of coagulation cascade activation, is associated with increased airway inflammation and hyperreactivity (44). Many allergens, including fungi such as *A. niger*, possess protease activities that are capable of cleaving fibrinogen, releasing fibrinogen cleavage products that, when introduced intranasally to mice, induce pulmonary inflammation and increase mucus secretion and bronchial hyperreactivity in a TLR4-dependent manner (13, 14). In CRS, upregulation of the coagulation cascade has been associated with the eosinophilic type 2 CRS subtype and shown to induce secretory activity in nasal polyp fibroblasts that contribute to the deposition of fibrin within the sinonasal mucosa (41, 45, 46). Similarly, in our microarray study comparing expression from 22 healthy controls and 120 CRS patients, genes associated with platelet activation and coagulation pathways were also upregulated in the inflamed sinus mucosa (47). Consistent with human CRS, our murine model of eosinophilic CRS also demonstrated the induction of genes associated with coagulation and platelet activation. Consequently, future studies can specifically interrogate the role of these pathways in the pathogenesis of these type 2 inflammatory diseases utilizing our murine model of concurrent eosinophilic CRS and allergic asthma.

In addition to its humoral aspect, the cellular aspect of the coagulation system, consisting of megakaryocytes and platelets, is also activated in the context of distinct allergenic fungi such as *Candida* spp., a group of similar fungal pathogens that is etiologically implicated in asthma and CRS and related disorders within the spectrum of unified airway disease (32, 48–51). In contrast to molds such as *A. niger, C. albicans* induces eosinophilic inflammation not through proteinases and fibrinogen, but rather through a distinct virulence factor, candidalysin (52), that activates platelets through the von Willebrand factor receptor GP1bα. Candidalysin-activated platelets release the Wnt-beta catenin pathway antagonist dickkopf-1 (Dkk-1) that coordinately promotes Th2 and Th17 differentiation that drives disease expression (15).

It remains unknown if *A. niger* and similar proteinases can also promote Dkk-1 release from platelets. However, Dkk-1 is implicated in bone destructive phenotypes and osteoclast activation that characterize autoimmune joint diseases such as rheumatoid arthritis (53, 54). As Dkk-1 plasma levels are also increased in asthma and CRS (15), the latter including clinically significant destructive changes of the bones surrounding involved sinuses (24, 55), our findings suggest that Dkk-1 could represent a factor common in mouse and human allergic disease contexts that explains the consistent link to osteoclast differentiation. Activated platelets also display enhanced mRNA processing activity resulting in distinct mRNA isoforms (alternative splicing) (56), a molecular feature common to other activated inflammatory cells linked to unified airway disease (57–62). These observations potentially explain the link between mouse and human allergic airway diseases and spliceosome activation. Additional studies are required to understand the airway mycosis-dependent signals that activate both osteoclast and splicesome-dependent pathways.

Many of the cytokines associated with type 2 inflammation including IL-4, IL-13, and thymic stromal lymphopoietin (TSLP) signal through the JAK-STAT pathway (63). Stimulated by both IL-4 and IL-13, IL-4Rα leads to the phosphorylation of Janus kinases and activation of STAT6. The IL-4/IL-13/STAT6 pathway induces expression of inflammatory chemokines such as CCL11, CXCL1, and CXCL3 and is a central pathway in the pathophysiology of asthma, especially airway hyperresponsiveness (8). As such, IL-4 and IL-13 signaling antagonists (e.g., dupilumab) have proven to be highly successful commercially, and several therapeutics targeting STAT6 directly, including small peptide inhibitors of the SH2 domain of STAT5 and STAT6 (10), have been evaluated and shown to be successful in preclinical studies of experimental asthma. The development and characterization of our fungus-induced mouse model of combined airways disease provide a critical resource to understanding the pathogenesis of fungus-induced airway inflammation and to evaluate the effects of novel therapeutic treatments such as a STAT6 small peptide inhibitor on either or both the upper and lower airway. Comparative pathway analysis of our mouse model of eosinophilic CRS with human CRS and asthma confirmed the expected induction of type 2 inflammation and JAK-STAT pathways. The reduction in sinus inflammation along with improvement in lower airway hyperreactivity with a topical STAT6 inhibitor with our mouse model provides promising preclinical support as a novel therapeutic option.

In summary, our results show that 3 months of intranasal exposure to fungal conidia leads simultaneously to eosinophilic sinus inflammation and allergic lower airway disease. Cellular, comparative sequencing, and pathway analyses highlight the overlap between this murine model and human CRS and asthma with respect to platelets and the coagulation system, the spliceosome, osteoclast activation, and numerous other regulatory pathways with potential links to fungal-induced inflammation. In addition to providing a unique platform for further understanding the immunologic basis of asthma and sinusitis, this model underscores the important role of environmental fungi as etiologic agents of unified airway disease.

## Data Availability Statement

The datasets presented in this study can be found in online repositories. The names of the repository/repositories and accession number(s) can be found below: Gene expression omnibus GSE193519.

## Ethics Statement

The animal study was reviewed and approved by the Institutional Animal Care and Use Committee of Baylor College of Medicine.

## Author Contributions

HS, DC, AL, and JK designed experiments, analyzed data, and supervised the project. EO, Y-DL, KP, YZ, and MC performed experiments and analyzed data. AD, MM, and JA collected samples and performed computational analysis. All authors contributed to the article and approved the submitted version.

## Funding

The content is solely the responsibility of the authors and does not necessarily represent the official views of the United States National Institutes of Health or the Veterans Administration Office of Research and Development. Supported by US National Institutes of Health grants R01HL117181, HL140398, R01AI135803, and R41AI124997 and the Department of Veterans Affairs Biomedical Laboratory Research and Development Merit Review Award I01BX004828. This project was further supported by the Cytometry and Cell Sorting Core at Baylor College of Medicine with funding from the CPRIT Core Facility Support Award (CPRIT-RP180672) and the NIH (CA125123 and RR024574), and the assistance of Joel M. Sederstrom.

## Conflict of Interest

DC and JK hold intellectual property in PM-43I.

The remaining authors declare that the research was conducted in the absence of any commercial or financial relationships that could be construed as a potential conflict of interest.

The handling editor declared a shared affiliation with the author YZ at the time of review.

## Publisher’s Note

All claims expressed in this article are solely those of the authors and do not necessarily represent those of their affiliated organizations, or those of the publisher, the editors and the reviewers. Any product that may be evaluated in this article, or claim that may be made by its manufacturer, is not guaranteed or endorsed by the publisher.

## Supplementary Material

Supplementary Figure 1 Intranasal fungal challenge promotes eosinophilic lower airway inflammation. **(A)** Mice were i.n. challenged with 4 × 10^5^ A. niger (AN) or PBS every other day for 2 weeks and assessed for changes in airway resistance (RRS). **(B)** H&E and **(C)** PAS staining of lung tissue from indicated challenge groups. Scale bars in the low and high magnification were 100μm and 10μm respectively. Results represent mean ± s.e.m. from two independent experiments; p < 0.001, n = 5 by two-way repeated measures ANOVA.

Supplementary Figure 2 Sorted sinus inflammatory cells. Mice were challenged with 4 x 10^5^ A*. niger* conidia for 2 months and sinus inflammatory cells were sorted.

Supplementary Table 1 Table lists published murine models of CRS, showing duration, route, allergen/adjuvant formulation, and pathology observed.

Supplementary Table 2 Table lists differentially expressed genes. Gene Ensembl ID, symbol, fold change, significance (*p*_adj_ < 0.05), full name and simplemine descriptions are presented in their corresponding columns.

## Abbreviations

AMP, antimicrobial peptide; AN, *Aspergillus niger*; CAMP, cationic antimicrobial peptide; CRS, chronic rhinosinusitis; CRSwNP, chronic rhinosinusitis with nasal polyps; DEGs, differentially expressed genes; Dkk-1, dickkopf-1; PAR-2, proteinase activated receptor 2; RNA-seq, RNA sequencing; TSLP, thymic stromal lymphopoietin.
