# Supplementary figures and images for: STAT6 Blockade Abrogates Aspergillus-Induced Eosinophilic Chronic Rhinosinusitis and Asthma, A Model of Unified Airway Disease
Source: Front Immunol. 2022 Feb 23;13:818017. doi: 10.3389/fimmu.2022.818017 (PMC8904741; doi:10.3389/fimmu.2022.818017)

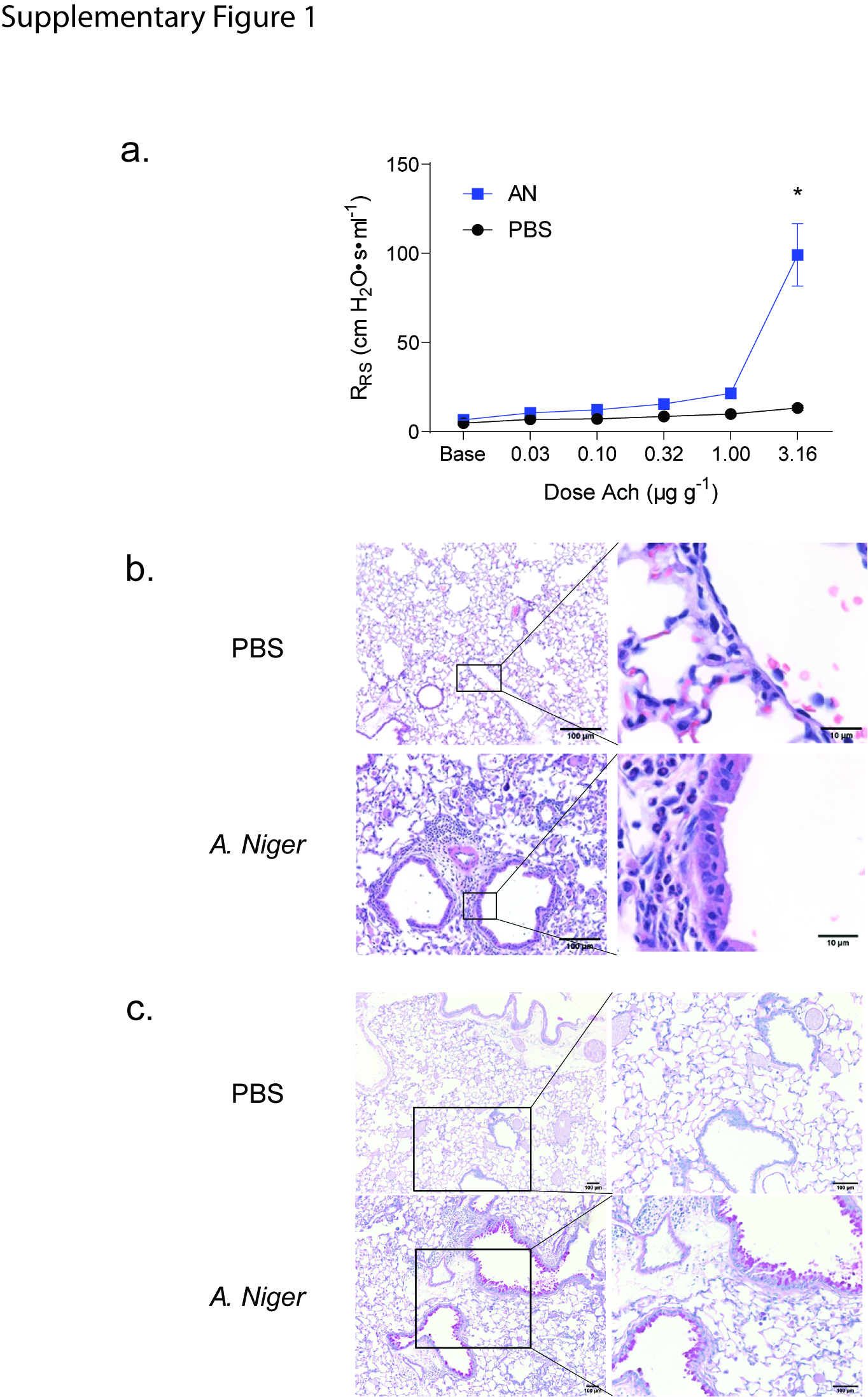

Supplement: Supplementary Figure 1 — Intranasal fungal challenge promotes eosinophilic lower airway inflammation. (A) Mice were i.n. challenged with 4 × 10^5^ A. niger (AN) or PBS every other day for 2 weeks and assessed for changes in airway resistance (RRS). (B) H&E and (C) PAS staining of lung tissue from indicated challenge groups. Scale bars in the low and high magnification were 100μm and 10μm respectively. Results represent mean ± s.e.m. from two independent experiments; p < 0.001, n = 5 by two-way repeated measures ANOVA. [file Image_1.tif]

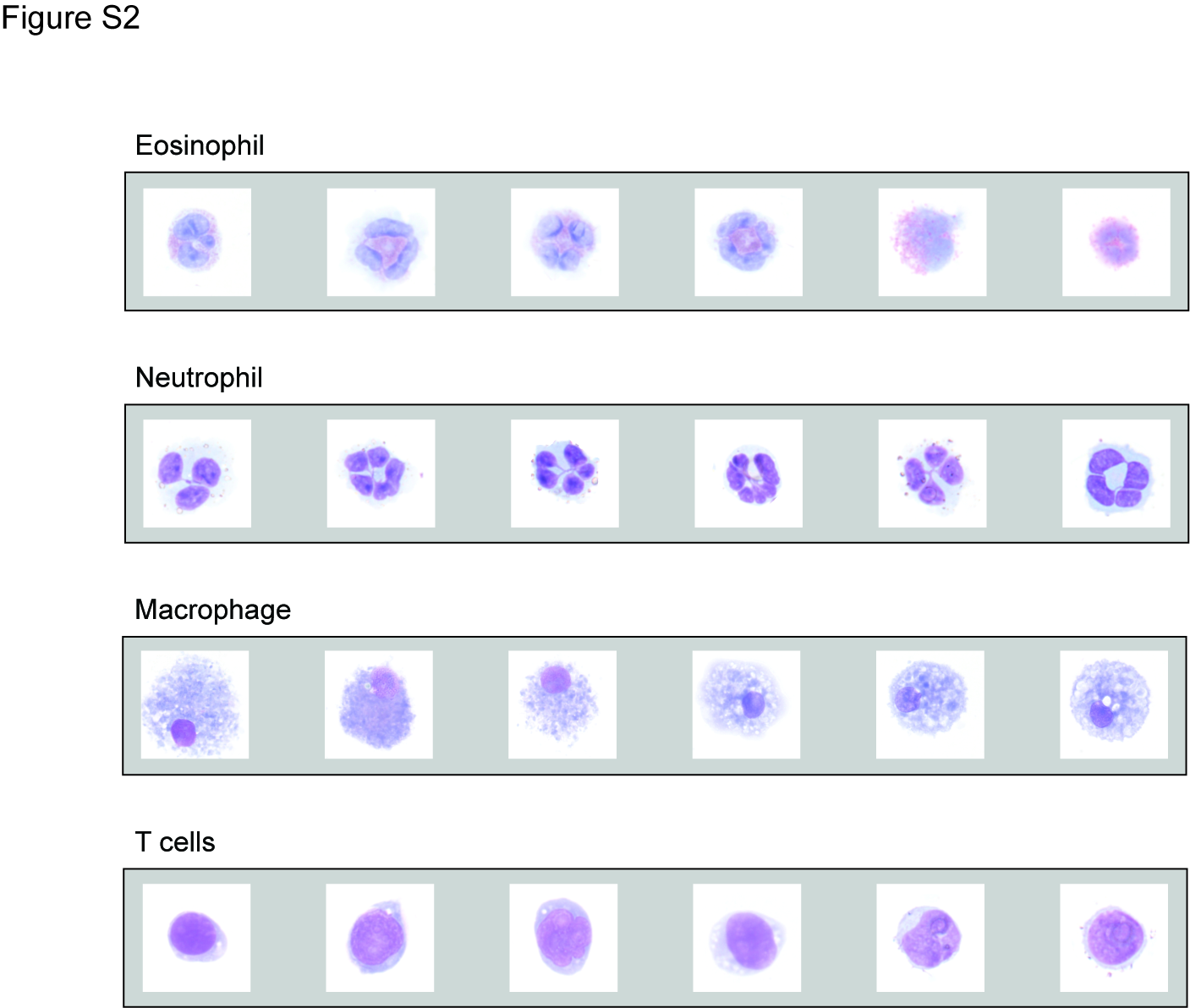

Supplement: Supplementary Figure 2 — Sorted sinus inflammatory cells. Mice were challenged with 4 x 10^5^ A. niger conidia for 2 months and sinus inflammatory cells were sorted. [file Image_2.tif]

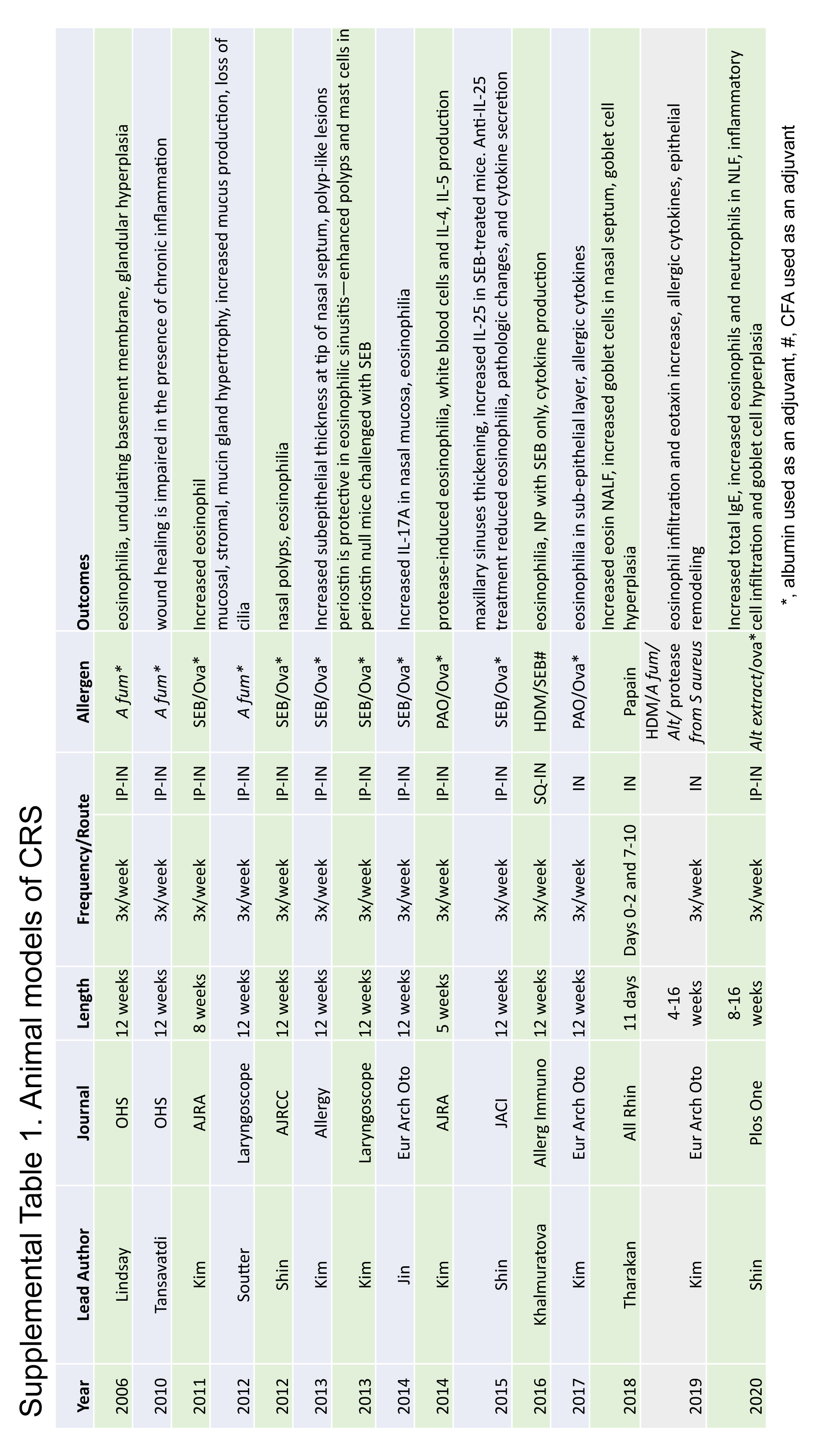

Supplement: Supplementary Table 1 — Table lists published murine models of CRS, showing duration, route, allergen/adjuvant formulation, and pathology observed. [file Image_3.tif]
